# Clinical efficacy and complications of MIS-TLIF and TLIF in the treatment of upper lumbar disc herniation: a comparative study

**DOI:** 10.1186/s13018-024-04806-9

**Published:** 2024-05-28

**Authors:** Bochen An, Bowen Ren, Yihao Liu, Zhenchuan Han, Jianhui Wu, Keya Mao, Jianheng Liu

**Affiliations:** 1 Medical School of Chinese PLA, Beijing, 100853 China; 2 Department of Orthopedics, Chinese PLA Rocket Force Characteristic Medical Center, Beijing, 100088 China; 3 Department of Orthopedics, Chinese PLA General Hospital, Beijing, 100853 China

**Keywords:** Intervertebral degenerative disease, Upper lumbar disc herniation, Minimally invasive transforaminal lumbar interbody fusion, Transforaminal lumbar interbody fusion

## Abstract

**Background:**

The optimal treatment modality for upper lumbar disc herniation remains unclear. Herein, we compared the clinical efficacy and application value of minimally invasive transforaminal lumbar interbody fusion (MIS-TLIF) and transforaminal lumbar interbody fusion (TLIF) for upper lumbar disc herniation. We aimed to provide new evidence to guide surgical decisions for treating this condition.

**Methods:**

We retrospectively analyzed the clinical data of 81 patients with upper lumbar disc herniation admitted between January 2017 and July 2018, including 41 and 40 patients who underwent MIS-TLIF and TLIF, respectively. Demographic characteristics, preoperative functional scores, perioperative indicators, and postoperative complications were compared. We performed consecutive comparisons of visual analog scale (VAS) scores of the lumbar and leg regions, Oswestry disability index (ODI), Japanese Orthopaedic Association scores (JOA), and MacNab scores at the final follow-up, to assess clinical outcomes 5 years postoperatively.

**Results:**

VAS scores of the back and legs were significantly lower in the MIS-TLIF than the TLIF group at 3 months and 1 year postoperatively (*P* < 0.05). Intraoperative bleeding and postoperative hospitalization time were significantly lower, and the time to return to work/normal life was shorter in the MIS-TLIF than in the TLIF group (*P* < 0.05). The differences in JOA scores and ODI scores between the two groups at 3 months, 1 year, and 3 years postoperatively were statistically significant (*P* < 0.05).

**Conclusion:**

The early clinical efficacy of MIS-TLIF was superior to that of TLIF, but no differences were found in mid-term clinical efficacy. Further, MIS-TLIF has the advantages of fewer medical injuries, shorter hospitalization times, and faster postoperative functional recovery.

## Background

Upper lumbar discs include the L1–2, L2–3, and L3–4 disc segments. Compared with the lower lumbar vertebrae, the incidence of upper lumbar disc herniation (ULDH) is significantly lower, accounting for approximately 1–3.8% of all patients with lumbar disc herniation [1]. The anatomical characteristics of the vertebral bodies and appendages of the upper lumbar vertebrae differ significantly from those of the lower lumbar vertebrae. The vertebral bodies and intervertebral discs of the upper lumbar vertebrae are smaller, and their spinal canal is mostly subtriangular or ovoid, with a shallow lateral recess, resulting in a smaller spinal canal than the lower lumbar vertebral canal [2]. In addition, the epidural space is smaller and has less epidural fat, while the surrounding anatomical environment lacks sufficient cushioning space, and there are relatively more nerves traveling in the dural sac. As such, a herniated disc in the upper lumbar spine will more likely compress more nerve tissues and complicate symptomatic manifestations [3]. Moreover, it has been shown that the development of ULDH is often accompanied by multifidus muscle degeneration [4]. Therefore, investigating means to decrease the likelihood of lumbar muscle injury in the treatment of ULDH should be prioritized. With the development and advancement of surgical techniques, the applications of minimally invasive transforaminal lumbar interbody fusion (MIS-TLIF) have expanded. Several clinical studies have confirmed that MIS-TLIF achieves comparable results to traditional intervertebral decompression and fusion in terms of postoperative clinical efficacy but has the advantages of less bleeding and less soft tissue damage [5, 6]. However, there are limited reports of studies related to MIS-TLIF for the treatment of ULDH. Moreover, no study has yet reported on the efficacy of MIS-TLIF versus the conventional TLIF approach for treating ULDH. Therefore, we retrospectively compared the postoperative clinical efficacy, complications, and patient satisfaction achieved by MIS-TLIF and conventional TLIF. We also summarized the specific advantages of MIS-TLIF in treating ULDH. The aim of this study was to provide new ideas for the treatment of ULDH.

## Methods

### Patients

This study comprised a retrospective analysis of 81 patients treated for lumbar disc herniation from January 2017 to July 2018. All patients were categorized into the MIS-TLIF and TLIF groups according to the surgical approach, with 41 and 40 patients in each group, respectively.

The inclusion criteria were: (1) computed tomography and magnetic resonance imaging showing central, paracentral, or prolapse of a single segment of the upper lumbar spine; (2) radicular calf pain consistent with imaging findings and failure of more than 3 months of extensive conservative treatment, including medications, physical therapy, and other treatments; (3) patients without segmental instability in plain images; (4) patients willing to undergo lumbar fusion internal fixation surgery and cooperate with regular follow-up visits. The exclusion criteria were: (1) history of lumbar spine surgery; (2) patients aged < 25 or > 80 years; (3) patients with a diagnosis of multi-segmental spinal disease; (4) preoperative comorbidities such as fresh fractures, infections, tumors, severe osteoporosis, rheumatoid arthritis, or other neurological diseases.

### Surgical techniques

**Fig. 1:**
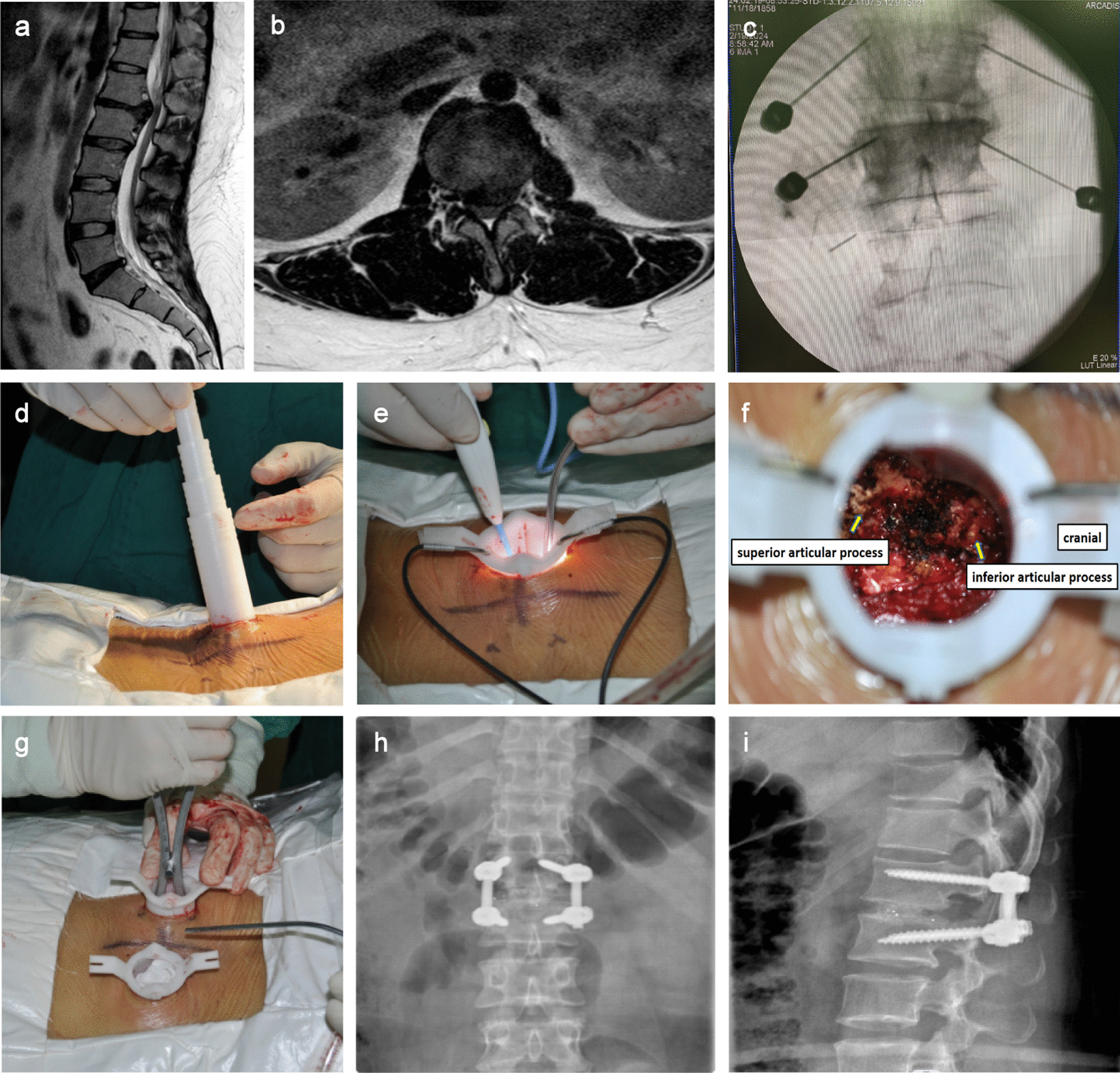
Patient, female, 26 years old, upper lumbar disc herniation undergoing MIS-TLIF surgery (L1-L2). **a** Patient's preoperative sagittal MRI. **b** Patient's preoperative horizontal MRI. **c** Intraoperative positioning of the long needle puncture. **d** Placement of disposable working tube. **e** the light source was placed and the soft tissues around the facet joint were eliminated. **f** Surgical view in the working tube. **g** On the symptomatic side, the same method was used as on the contralateral side to place the tube, prepare the nail paths, and seal it with bone wax. **h**, **i** It was satisfactory to determine the position of the rod and cage on postoperative fluoroscopy

For the MIS-TLIF approach, the patient was placed in the prone position under general anesthesia. On the non-symptomatic side of the posterior midline, a 3-cm paracentesis was made, and two 9-gauge long needles were positioned, using fluoroscopy to adjust the needle tip position. Along the line between the two needles, the skin, subcutaneous tissue, and deep fascia were sequentially incised, and the muscle space was bluntly separated to reach the facet joint. The disposable working tube was placed and propped open after step-by-step dilatation; a light source was placed and the soft tissues around the facet joint were eliminated. Afterward, the nail paths were prepared at the upper and lower pedicles by applying the transverse process localization method. When the nail paths were correctly positioned under fluoroscopy, the pedicle screw was inserted. The pre-curved titanium rods were inserted in turn, appropriately propped up, and lifted to reset the position, and the locking nut was tightened. On the symptomatic side, the same method was used as on the contralateral side to place the tube, prepare the nail paths, and seal them with bone wax. Subsequently, the superior articular process, a portion of the lamina, the inferior articular process, and the hyperplastic ligamentum flavum were removed. After implanting the PEEK material cage filled with autogenous bone particles into the intervertebral space, the pedicle screws were screwed into the pre-determined nail channels, and titanium rods were installed for pressure fixation. The position of the rods and the cage were determined to be satisfactory under C-arm fluoroscopy. No drainage was placed, and the incision was closed one layer at a time after rinsing to complete the surgery (Fig. 1).

For the conventional TLIF operation, we performed a longitudinal incision in the posterior midline, peeled off the paraspinal muscles, inserted pedicle screws, resected the vertebral lamina, and performed facetectomy on the symptomatic side. After the nucleus pulposus was removed and the vertebral endplate cartilage was scraped, the PEEK material cage filled with autologous bone particles was implanted into the intervertebral space and titanium rods were installed. Finally, a drain was placed to complete the operation. All surgeries were performed by the same senior surgeon. Antibiotics, dehydrating agents, and neurotrophic drugs were administered postoperatively to the patients in both groups. The brace was worn on the third day after surgery to assist with mobility for 3 months.

### Clinical assessments and follow-up

The clinical indicators of patients in both groups were recorded and analyzed, including operation time, intraoperative blood loss, postoperative complications, and postoperative hospitalization time. The patients were followed up by telephone or clinical follow-up at 3 months and 1, 3, and 5 years after discharge. The Oswestry disability index (ODI) score, Visual analog scale (VAS) score, and Japanese Orthopaedic Association scores (JOA) of lumbar pain and sciatica were recorded at each follow-up. Postoperative clinical outcomes were evaluated based on the modified MacNab scores.

### Statistical analysis

All experimental data were statistically analyzed using IBM SPSS software (version 26.0, IBM Corp., Armonk, NY, USA). Normally distributed measures were expressed as mean ± standard deviation (SD), and skewed measures were expressed as median ± interquartile range (IQR). Comparisons between groups of measures that were normally distributed with homoscedasticity were made using independent samples t-test. Comparisons between groups of measures that were skewed or heteroscedastic were made using the Mann–Whitney rank-sum test. The χ^2^ and Mann–Whitney rank-sum tests were used to compare unordered and ordered qualitative data, respectively. Differences were considered statistically significant at *P* < 0.05.

## Results

### Demographic and clinical information

A total of 81 patients were included in this study. Of the 41 patients in the MIS-TLIF group, 20 were men, and 21 were women, with a mean age of 54.0 ± 11.8 years, a mean body mass index of 27.0 ± 4.0 kg/m^2^, and a mean symptom duration of 6.00 (5.00,7.00) months. In total, 7.3%, 26.8%, and 65.9% of patients in the MIS-TLIF group developed lumbar disc herniation in L1–L2, L2–L3, and L3–L4, respectively. The incidence of lumbar disc herniation in the TLIF group was 15%, 22.5%, and 62.5% at L1–L2, L2–L3, and L3–L4, respectively. No significant differences existed between the MIS-TLIF and TLIF groups in terms of age, sex, body mass index, symptom duration, and operative segment. Detailed demographic and clinical information of the two groups are presented in Table 1.

**Table 1:** Demographic and clinical characteristics of patients in the two groups

	MIS-TLIF group (N = 41)	TLIF group (N = 40)	t/*z*/χ^2^ value	*P* value
Age (years)	54.0 ± 11.8	53.2 ± 10.3	0.326	0.746
Gender (female/male)	20/21	26/14	2.171	0.141
BMI (kg/m^2^)	27.0 ± 4.0	26.0 ± 4.4	1.058	0.293
Symptom duration (months)	6.00 (5.00,7.00)	6.50 (5.00,8.00)	− 1.176	0.240
Operative level			1.249	0.539
L1–L2	3	6		
L2–L3	11	9		
L3–L4	27	25		
Follow-up time (months)	65.6 ± 3.1	65.1 ± 2.7	− 0.675	0.500

Normally distributed data are expressed as mean ± SD and non-normally distributed data are expressed as the median (interquartile range)

*BMI* Body mass index, *MIS-TILF* Minimally invasive lumbar interbody fixation and fusion, *TILF* Transforaminal approach lumbar interbody fusion

### Perioperative parameters and complications

All 81 patients underwent surgery to treat single-segment ULDH, and the follow-up time was > 5 years, with no significant difference between the groups (*P* = 0.500). The average operation time in the MIS-TLIF group was 160.00 (135.00, 187.00) min, and that in the PLIF group was 152.00 (133.50, 177.25) min, with no significant difference between the two groups (*P* = 0.511). Intraoperative blood loss in the MIS-TLIF group was significantly lower than that in the TLIF group (*P* < 0.001), as was the mean postoperative hospitalization time (*P* < 0.001). Both MIS-TLIF and TLIF groups had one case of postoperative sensory disturbance each, and the symptoms were relieved in both cases by nerve nutrition. In the TLIF group, there were two cases of poor wound healing, in which the wounds were considered infected; the patients had good wound healing after two weeks of conservative treatment, including anti-infection and daily dressing change. There was no fracture or loosening of the endoprosthetic fixation, or displacement of the cage in either group, and the difference in complication rates between the two groups was not statistically significant (*P* = 0.359, Table 2).

**Table 2:** Operation parameters and complications experienced by patients in the two groups

	MIS-TLIF group (N = 41)	TLIF group (N = 40)	*z*/χ^2^ value	*P* value
Operation time (min)	160.00 (135.00,187.00)	152.50 (133.50,177.25)	− 0.657	0.511
Estimated blood loss (mL)	100.00 (50.00,100.00)	100.00 (100.00,150.00)^*^	− 3.447	0.001^*^
Postoperative hospitalization stay (days)	4.00 (3.00,5.00)	5.00 (4.00,6.00)^*^	− 3.902	0.000^*^
Complications	1	3	–	0.359
Dural tear	0	0		
Postoperative dysesthesia	1	1		
Poor wound healing	0	2		
Hematoma	0	0		
Infection	0	0		

Values are expressed as the median (interquartile range). *P < 0.05

*MIS-TLIF* Minimally invasive lumbar interbody fixation and fusion, *TLIF* Transforaminal approach lumbar interbody fusion

### Therapeutic effects

The lumbar VAS scores in the MIS-TLIF group were significantly lower than those in the TLIF group at the 3-month, 1-year, and 3-year postoperative follow-ups (*P* < 0.05). Further, the lumbar VAS scores in the MIS-TLIF group were slightly lower than those in the TLIF group at the 5-year postoperative follow-up, but this difference was not statistically significant (*P* > 0.05). The leg VAS scores in the MIS-TLIF group were lower than those in the TLIF group at the 3-month postoperative and 1-year postoperative follow-ups and were significantly different (*P* < 0.05), and the leg VAS scores in the MIS-TLIF group were lower than those in the TLIF group at the 3-year postoperative and 5-year postoperative follow-ups, although not significantly (*P* > 0.05). The ODI scores in the MIS-TLIF group were lower than those in the TLIF group at 3 months, 1 year, and 3 years postoperatively (*P* < 0.05). The ODI scores in the MIS-TLIF group were lower than those in the TLIF group at 5 years postoperatively, although not statistically significant (*P* > 0.05). The JOA scores in the MIS-TLIF group were higher than those in the TLIF group at the 3-month, 1-year, and 3-year postoperative follow-ups (*P* < 0.05). Although this trend continued at 5 years postoperatively, it was not statistically significant (*P* > 0.05). The modified MacNab scores in the MIS-TLIF group were excellent in 23 cases (56.1%), good in 17 cases (41.5%), and fair in one case (2.4%), while the results in the TLIF group were excellent in 19 cases (47.5%), good in 14 cases (35%), fair in 6 cases (15%), and poor in one case (2.5%). The distribution of the MacNab criterion assessment was not significantly different between the two groups (*P* = 0.146) (Table 3).

**Table 3:** Therapeutic effects and modified MacNab criterion assessments of the two groups

	MIS-TLIF group (N = 41)	TLIF group (N = 40)	*z*/χ^2^ value	*P* value
*VAS back pain*				
Preoperative	8.00 (7.00, 8.00)	7.50 (7.00, 8.00)	− 0.694	0.488
Postoperative 3 months	3.00 (2.00, 3.00)	4.00 (3.00, 4.00)^*^	− 5.139	0.000^*^
Postoperative 1 years	2.00 (1.50, 2.50)	2.00 (2.00, 3.00)^*^	− 2.356	0.018^*^
Postoperative 3 years	1.00 (1.00, 2.00)	2.00 (2.00, 3.00)^*^	− 2.240	0.025^*^
Postoperative 5 years	1.00 (0.00, 2.00)	2.00 (1.00, 2.00)	− 1.658	0.097
*VAS leg pain*				
Preoperative	7.00 (6.00, 8.00)	6.00 (6.00, 7.75)	− 1.782	0.075
Postoperative 3 months	2.00 (1.00, 2.00)	2.00 (2.00, 3.00)^*^	− 2.376	0.017^*^
Postoperative 1 years	1.00 (1.00, 1.50)	2.00 (1.00, 3.00)^*^	− 3.048	0.002^*^
Postoperative 3 years	1.00 (0.00, 1.00)	1.00 (0.00, 2.00)	− 0.863	0.388
Postoperative 5 years	0.00 (0.00, 1.00)	1.00 (0.00, 1.00)	− 0.504	0.614
*ODI scores*				
Preoperative	28.00 (25.00, 34.00)	26.50 (25.00, 28.00)	− 1.533	0.125
Postoperative 3 months	14.00 (11.50, 15.00)	15.00 (14.00, 16.00)^*^	− 2.253	0.024^*^
Postoperative 1 years	8.00 (7.00, 11.00)	10.00 (8.00, 12.00)^*^	− 2.161	0.031^*^
Postoperative 3 years	5.00 (3.50, 8.00)	6.00 (4.00, 10.75)^*^	− 2.107	0.035^*^
Postoperative 5 years	5.00 (3.00, 7.50)	5.00 (3.25, 9.00)	− 1.594	0.111
*JOA scores*				
Preoperative	8.00 (5.00, 10.00)	9.00 (8.00, 10.00)	− 1.816	0.069
Postoperative 3 months	16.00 (14.50, 19.00)	14.00 (13.00, 15.75)^*^	− 4.614	0.000^*^
Postoperative 1 years	19.00 (17.50, 21.00)	18.00 (16.00, 19.00)^*^	− 2.460	0.014^*^
Postoperative 3 years	23.00 (20.50, 25.00)	21.00 (19.00, 23.00)^*^	− 3.087	0.002^*^
Postoperative 5 years	24.00 (21.00, 25.00)	22.00 (20.00, 24.00)	− 1.893	0.058
*Modified MacNab*			5.014	0.146
Excellence	23	19		
Good	17	14		
Fair	1	6		
Poor	0	1		

Values are expressed as the median (interquartile range). *P < 0.05

*JOA* Japanese Orthopaedic Association scores, *MIS-TLIF* Minimally invasive transforaminal lumbar interbody fusion, *ODI* Oswestry disability index, *TLIF* Transforaminal lumbar interbody fusion, *VAS* Visual analog scale

## Discussion

The upper lumbar spine has a narrower spinal canal than the lower lumbar spine [2]. When ULDH occurs, the lumbar and cauda equina nerve roots are more prone to compression. Owing to its anatomical structure, the small cushioning space of the spinal canal often makes it difficult to treat the symptoms because of compression severity [7]. Moreover, because the nerve roots that travel through the upper lumbar spine do not innervate any specific muscles, ULDH can lead to nonspecific clinical symptoms and neurological manifestations, which may lead to misdiagnosis or underdiagnosis of ULDH [8]. Therefore, early diagnosis and timely surgical treatment to relieve the cauda equina and nerve root compression are key to treating this disease. Owing to the unique characteristics of the upper lumbar vertebrae, traditional lumbar discectomy often results in suboptimal surgical outcomes for ULDH. Furthermore, for patients with central lumbar disc herniation or those with instability in the affected segment, lumbar discectomy often fails to remove the herniated disc completely and alleviate symptoms [9]. Therefore, in such cases, lumbar spinal fusion surgery may be a more suitable option. TLIF is a well-established technique that can reduce the damage caused by intraoperative nerve root pulling in treating lumbar disc herniation and improve neurogenic symptoms. However, peeling off the muscle to the vertebral plate layer by layer through the incision can destroy the biomechanical structure of the paraspinal muscles and affect the overall stability of the lumbar vertebrae [10]. In contrast, MIS-TLIF involves the removal of the articular synovial joint and part of the lamina through a multifidus interspace approach, preserving the structure of the musculoligamentous complex, thereby reducing the risk of injury to the paraspinal muscles and nerves [11]. However, the MIS-TLIF technique has been suggested to increase the risk of inadequate nerve decompression because of the limited intraoperative field of view, leading to deficiencies in surgical and long-term clinical outcomes [12]. Currently, there is no definitive conclusion regarding the optimal surgical modality for treating ULDH. This study is the first to compare the clinical efficacy of MIS-TLIF with that of TLIF for treating ULDH at different time points after surgery.

Many studies have shown that MIS-TLIF has a longer operative time than open surgery, which is attributed to the steep learning curve of MIS-TLIF and the need for additional intraoperative fluoroscopy to optimize the position of the pedicle screws [13, 14]. Price et al. investigated 452 patients who underwent TLIF surgery and reported that the MIS-TLIF procedure time was significantly lower than that of TLIF [15]. However, in this study, the difference in operative time between MIS-TLIF and TLIF was not statistically significant (*P* > 0.05). This was possibly because of the relatively narrow anatomical environment of the upper lumbar spine for the MIS-TLIF surgical operation process. This process requires more time to expose the dural sac, clarify the location of the nerve root, perform careful percutaneous placement of screws, and ensure no nerve damage, followed by successful completion of decompression and fusion of the intervertebral disc. In addition, some studies now show that MIS-TLIF is less than TLIF in terms of intraoperative blood loss [14, 16]. In the present study, intraoperative bleeding in the MIS-TLIF group was 100.0 (50.0, 100.0) mL, which was significantly lower than that in the TLIF group, 100 (100.0, 150.0) mL (*z* = − 3.447; *P* < 0.001). This is consistent with the results of the meta-analysis by Hammad et al. [17]. The initial complication rate of MIS-TLIF has been reported to range from 6.8 to 23.8%, with the surgical complication rate decreasing as the technical curve gradually flattens out [18]. Here, no significant complications in either group were encountered. However, there was one case of postoperative sensory impairment in MIS-TLIF, considered a reversible nerve injury caused by prolonged compression of the upper lumbar intervertebral disc. The patient experienced symptomatic relief after receiving neurotrophic medication for 3 months. No significant difference in the incidence of common postoperative complications between the MIS-TLIF and TLIF groups (*P* = 0.359) was observed, consistent with previous reports [17]. Most studies have concluded that increased intraoperative blood loss is significantly associated with longer postoperative hospital stays [16]. Indeed, in the present study, the length of postoperative hospitalization in the MIS-TLIF group was significantly shorter than that in the TLIF group (*P* < 0.001), which implies that this technique can reduce the costs of the hospitalization process [19]. This was consistent with the results reported by Zhang et al. [20]. The ERAS concept, introduced by Kehlet in 1997, is important in perioperative management aimed at improving the functional recovery of patients while reducing the length of hospitalization, incidence of postoperative complications, and early return to a normal social life [21]. MIS-TLIF to treat ULDH meets these criteria, as the shorter surgical incisions and less muscle and soft tissue damage result in less postoperative bleeding and shorter postoperative hospitalization.

The VAS, JOA, and ODI scores are commonly used to assess the functional recovery of the lumbar spine and postoperative outcomes [22–24]. Most studies have shown that MIS-TLIF causes less postoperative damage to the paraspinal muscles and soft tissues than traditional TLIF, significantly reducing patients’ lower back and leg VAS scores and ensuring better ODI functional scores [25]. However, no significant difference in postoperative VAS and ODI scores between patients treated with MIS-TLIF and TLIF has been shown [17]. In this study, the MIS-TLIF group had significantly lower back and leg VAS scores than the TLIF group at 3 months, 1 year, and 3 years postoperatively, consistent with previous studies [26]. The JOA and ODI scores in the MIS-TLIF group were significantly better than those in the TLIF group at 3 months, 1 year, and 3 years postoperatively. However, at 5 years postoperatively, there were no significant differences in the JOA and ODI scores between the two groups. It may be due to the fact that in the early postoperative period, the smaller skin incision and less mechanical damage to the paravertebral muscles of the MIS-TLIF protects the associated blood supply to the surrounding tissues [27]. This suggests that MIS-TLIF achieves more significant pain reduction and more rapid functional recovery in the short-term postoperative period, having better clinical efficacy. In this study, the postoperative efficacy of the two groups of patients was measured using the modified MacNab criteria. The efficacy rate of the MIS-TLIF group was excellent (97.6%), but there was no significant difference between the groups (*P* = 0.146). This suggests that both MIS-TLIF and TLIF can improve clinical symptoms in patients with ULDH.

Our study revealed the short- and mid-term clinical efficacy, advantages, and disadvantages of MIS-TLIF and TLIF in treating ULDH, which is clinically significant. Nevertheless, some limitations to this study should be mentioned. First, this was a retrospective study where some memory bias in patient selection could have existed. Second, the follow-up time was insufficient, and further follow-up is needed to compare the differences in long-term clinical efficacy between the two groups. In addition, all patients were treated by the same surgeon, limiting the generalizability of this study. Finally, the low prevalence of ULDH resulted in a small sample size. Therefore, prospective randomized controlled trials with larger sample sizes are needed to further determine the surgical advantages of MIS-TLIF for treating ULDH.

## Conclusions

MIS-TLIF and TLIF achieved excellent surgical results in treating ULDH; however, MIS-TLIF has the advantages of less intraoperative bleeding and shorter postoperative hospitalization than TLIF. No significant differences in operative time and postoperative complications between the two surgeries were observed. In terms of clinical efficacy, MIS-TLIF had superior JOA and ODI functional scores and VAS scores for the lower back and legs in the short-term postoperative period. In conclusion, this study suggest that MIS-TLIF is a reliable surgical procedure for the treatment of ULDH.

## Data Availability

The datasets used and/or analyzed during the current study are available from the corresponding author on reasonable request.

## Abbreviations

ULDH: Upper lumbar disc herniation
MIS-TLIF: Minimally invasive transforaminal lumbar interbody fusion
TLIF: Transforaminal lumbar interbody fusion
VAS: Visual analog scale
ODI: Oswestry disability index
JOA: Japanese Orthopaedic Association scores

## References

[1] Yüce I, Kahyaoğlu O, Mertan P, Çavuşoğlu H, Aydin Y (2019). Analysis of clinical characteristics and surgical results of upper lumbar disc herniations. Neurochirurgie.

[2] Lee DS, Park KS, Park MS (2013). The comparative analysis of clinical characteristics and surgical results between upper and lower lumbar disc herniations. J Korean Neurosurg Soc.

[3] Moon KH, Lee SH, Kong BJ, Shin SW, Bhanot A, Kim DY (2006). An oblique paraspinal approach for intracanalicular disc herniations of the upper lumbar spine: a technical case report. Neurosurgery.

[4] Liu C, Xue J, Liu J, Ma G, Moro A, Liang T (2021). Is there a correlation between upper lumbar disc herniation and multifidus muscle degeneration? A retrospective study of MRI morphology. BMC Musculoskelet Disord.

[5] Li F, Li C, Xi X, Zeng Z, Ma B, Xie N (2020). Distinct fusion intersegmental parameters regarding local sagittal balance provide similar clinical outcomes: a comparative study of minimally invasive *versus* open transforaminal lumbar interbody fusion. BMC Surg.

[6] Zhao J, Zhang S, Li X, He B, Ou Y, Jiang D (2018). Comparison of minimally invasive and open transforaminal lumbar interbody fusion for lumbar disc herniation: a retrospective cohort study. Med Sci Monit.

[7] Hioki A, Miyamoto K, Hosoe H, Sugiyama S, Suzuki N, Shimizu K (2011). Cantilever transforaminal lumbar interbody fusion for upper lumbar degenerative diseases (minimum 2 years follow up). Yonsei Med J.

[8] Bae J, Lee SH, Shin SH, Seo JS, Kim KH, Jang JS (2016). Radiological analysis of upper lumbar disc herniation and spinopelvic sagittal alignment. Eur Spine J.

[9] Yuan C, Wang J, Zhou Y, Pan Y (2018). Endoscopic lumbar discectomy and minimally invasive lumbar interbody fusion: a contrastive review. Wideochir Inne Tech Maloinwazyjne.

[10] Erdoğan U (2021). The results of using a transforaminal lumbar interbody fusion cage at the upper lumbar level. Cureus.

[11] Kim CH, Easley K, Lee JS, Hong JY, Virk M, Hsieh PC (2020). Comparison of minimally invasive *versus* open transforaminal interbody lumbar fusion. Glob Spine J.

[12] Hey HW, Hee HT (2010). Lumbar degenerative spinal deformity: surgical options of PLIF. TLIF MI-TLIF Indian J Orthop.

[13] Gu S, Li H, Wang D, Dai X, Liu C (2022). Application and thinking of minimally invasive transforaminal lumbar interbody fusion in degenerative lumbar diseases. Ann Transl Med.

[14] Seng C, Siddiqui MA, Wong KP, Zhang K, Yeo W, Tan SB (2013). Five-year outcomes of minimally invasive *versus* open transforaminal lumbar interbody fusion: a matched-pair comparison study. Spine (Phila Pa 1976).

[15] Price JP, Dawson JM, Schwender JD, Schellhas KP (2018). Clinical and radiologic comparison of minimally invasive surgery with traditional open transforaminal lumbar interbody fusion: a review of 452 patients from a single center. Clin Spine Surg.

[16] Ao S, Zheng W, Wu J, Tang Y, Zhang C, Zhou Y (2020). Comparison of preliminary clinical outcomes between percutaneous endoscopic and minimally invasive transforaminal lumbar interbody fusion for lumbar degenerative diseases in a tertiary hospital: is percutaneous endoscopic procedure superior to MIS-TLIF? A prospective cohort study. Int J Surg.

[17] Hammad A, Wirries A, Ardeshiri A, Nikiforov O, Geiger F (2019). Open *versus* minimally invasive TLIF: literature review and meta-analysis. J Orthop Surg Res.

[18] Shuman WH, Baron RB, Neifert SN, Martini ML, Chapman EK, Schupper AJ (2022). MIS-TLIF procedure is improving with experience: systematic review of the learning curve over the last decade. Clin Spine Surg.

[19] Parker SL, Mendenhall SK, Shau DN, Zuckerman SL, Godil SS, Cheng JS (2014). Minimally invasive *versus* open transforaminal lumbar interbody fusion for degenerative spondylolisthesis: comparative effectiveness and cost-utility analysis. World Neurosurg.

[20] Zhang H, Chen ZX, Sun ZM, Jiang C, Ni WF, Lin Y (2017). Comparison of the total and hidden blood loss in patients undergoing open and minimally invasive transforaminal lumbar interbody fusion. World Neurosurg.

[21] Debono B, Wainwright TW, Wang MY, Sigmundsson FG, Yang MMH, Smid-Nanninga H (2021). Consensus statement for perioperative care in lumbar spinal fusion: enhanced Recovery after Surgery (ERAS®) society recommendations. Spine J.

[22] Chiarotto A, Maxwell LJ, Ostelo RW, Boers M, Tugwell P, Terwee CB (2019). Measurement properties of visual analogue scale, numeric rating scale, and pain severity subscale of the brief pain inventory in patients with low back pain: a systematic review. J Pain.

[23] Haro H, Ebata S, Inoue G, Kaito T, Komori H, Ohba T (2022). Japanese Orthopaedic Association (JOA) clinical practice guidelines on the management of lumbar disc herniation, third edition – secondary publication. J Orthop Sci..

[24] Saberi H, Isfahani AV (2008). Higher preoperative Oswestry Disability Index is associated with better surgical outcome in upper lumbar disc herniations. Eur Spine J.

[25] Choi WS, Kim JS, Ryu KS, Hur JW, Seong JH (2016). Minimally invasive transforaminal lumbar interbody fusion at L5–S1 through a unilateral approach: technical feasibility and outcomes. BioMed Res Int.

[26] Phan K, Rao PJ, Kam AC, Mobbs RJ (2015). Minimally invasive *versus* open transforaminal lumbar interbody fusion for treatment of degenerative lumbar disease: systematic review and meta-analysis. Eur Spine J.

[27] Bhamb N, Kanim LEA, Maldonado RC, Nelson TJ, Salehi K, Glaeser JD (2019). The impact of type 2 diabetes on bone metabolism and growth after spinal fusion. Spine J.

